# CRITICAL ANALYSIS OF A PRAGMATIC TRIAL TO IMPROVE CHRONIC PAIN MANAGEMENT IN A WELFARE STATE

**DOI:** 10.1093/geroni/igae098.2494

**Published:** 2024-12-31

**Authors:** Dagmar Draeger, Daniela Koios, Arlett Wenzel, Andrea Budnick

**Affiliations:** Charité - Universitätsmedizin Berlin, Berlin, Berlin, Germany; Charité - Universitätsmedizin Berlin, Berlin, Berlin, Germany; Charité - Universitätsmedizin Berlin, Berlin, Berlin, Germany; Charité - Universitätsmedizin Berlin, Berlin, Berlin, Germany

## Abstract

High pain intensity and substantial pain interferences impact older community-dwelling adults with a certified need for care, while their pain management is not up to standard. A three-arm cluster-randomized pragmatic trial was conducted, with individualized interventions (IG1), digital staff training (IG2), and a control group (CG). The target group was home-care recipients (aged ≥65 years) with chronic pain. We recruited 190 participants from 22 home care providers (=clusters), drop-out rate was 24%. An expert panel communicated individualized nursing and medical recommendations for each IG1-participant. Nurses and physicians in IG2 were offered digital training. CG-participants received standard care. A dichotomous assessment regarding the acceptability of each participant’s pain situation is the primary outcome for this analysis (group differences assessed by χ²-tests). At baseline, 48.4% of participants described their pain situation as “not acceptable”. At follow-up, 15.9% stated an improvement of their pain situation, while the majority (70.7%) indicated stagnation or worsening (12.4%). There are no differences between the three study-arms. Staff commitment regarding intervention implementation did not meet the pre-defined requirements and varied substantially. Physicians and nursing staff cited heavy workloads, staff shortages etc. as arguments for not implementing interventions. While executives’ support is essential, commitment of those that are supposed to carry out interventions or changes is paramount. Implementing improved pain management in a pragmatic setting encountered structural obstacles in the German health system. When planning pragmatic trials, barriers to implementing interventions under real-world conditions must be critically discussed. We recommend involving required staff early in the planning process.

